# PEDF, a pleiotropic WTC-LI biomarker: Machine learning biomarker identification and validation

**DOI:** 10.1371/journal.pcbi.1009144

**Published:** 2021-07-21

**Authors:** George Crowley, James Kim, Sophia Kwon, Rachel Lam, David J. Prezant, Mengling Liu, Anna Nolan

**Affiliations:** 1 Department of Medicine, Division of Pulmonary, Critical Care and Sleep Medicine, New York University School of Medicine, New York, New York, United States of America; 2 Bureau of Health Services, Fire Department of New York, Brooklyn, New York, United States of America; 3 Department of Medicine, Pulmonary Medicine Division, Montefiore Medical Center and Albert Einstein College of Medicine, Bronx, New York, United States of America; 4 Department of Environmental Medicine, New York University School of Medicine, New York, New York, United States of America; 5 Department of Population Health, Division of Biostatistics, New York University School of Medicine, New York, New York, United States of America; University of Chicago, UNITED STATES

## Abstract

Biomarkers predict World Trade Center-Lung Injury (WTC-LI); however, there remains unaddressed multicollinearity in our serum cytokines, chemokines, and high-throughput platform datasets used to phenotype WTC-disease. To address this concern, we used automated, machine-learning, high-dimensional data pruning, and validated identified biomarkers. The parent cohort consisted of male, never-smoking firefighters with WTC-LI (FEV_1, %Pred_< lower limit of normal (LLN); n = 100) and controls (n = 127) and had their biomarkers assessed. Cases and controls (n = 15/group) underwent untargeted metabolomics, then feature selection performed on metabolites, cytokines, chemokines, and clinical data. Cytokines, chemokines, and clinical biomarkers were validated in the non-overlapping parent-cohort via binary logistic regression with 5-fold cross validation. Random forests of metabolites (n = 580), clinical biomarkers (n = 5), and previously assayed cytokines, chemokines (n = 106) identified that the top 5% of biomarkers important to class separation included pigment epithelium-derived factor (PEDF), macrophage derived chemokine (MDC), systolic blood pressure, macrophage inflammatory protein-4 (MIP-4), growth-regulated oncogene protein (GRO), monocyte chemoattractant protein-1 (MCP-1), apolipoprotein-AII (Apo-AII), cell membrane metabolites (sphingolipids, phospholipids), and branched-chain amino acids. Validated models via confounder-adjusted (age on 9/11, BMI, exposure, and pre-9/11 FEV_1, %Pred_) binary logistic regression had AUC_ROC_ [0.90(0.84–0.96)]. Decreased PEDF and MIP-4, and increased Apo-AII were associated with increased odds of WTC-LI. Increased GRO, MCP-1, and simultaneously decreased MDC were associated with decreased odds of WTC-LI. In conclusion, automated data pruning identified novel WTC-LI biomarkers; performance was validated in an independent cohort. One biomarker—PEDF, an antiangiogenic agent—is a novel, predictive biomarker of particulate-matter-related lung disease. Other biomarkers—GRO, MCP-1, MDC, MIP-4—reveal immune cell involvement in WTC-LI pathogenesis. Findings of our automated biomarker identification warrant further investigation into these potential pharmacotherapy targets.

## Author summary

Disease related to air pollution causes millions of deaths annually. Large swathes of the general population, as well as certain occupations such as 1^st^ responders and military personnel, are exposed to particulate matter (PM)—a major component of air pollution. Our longitudinal cohort of FDNY firefighters exposed to the World Trade Center dust cloud on 9/11 is a unique research opportunity to characterize the impact of a single, intense PM exposure by looking at pre- and post-exposure phenotype; however, PM-related lung disease and PM’s systemic effects are complex and call for a systems biological approach coupled with novel computational modelling techniques to fully understand pathogenesis. In the present study, we integrate clinical and environmental biomarkers with the serum metabolome, cytokines, and chemokines to develop a model for early disease detection and identification of potential signaling cascades of PM-related chronic lung disease.

## Introduction

Globally, air pollution (of which particulate matter [PM] is a significant component) contributes to pulmonary and vascular disease yielding a devastating 7 million annual deaths.[1–9] Lung injury due to inhalational exposure is a major health concern not only for 1^st^ responders and military personnel but also for large swaths of the population.[10,11] PM is a significant component of ambient air pollution and prominent in WTC-PM exposure.[12] The destruction of the World Trade Center (WTC) complex pulverized 1.2 million tons of construction material.[13,14] Particulate analysis showed that metals, such as chromium, nickel, and iron, powdered concrete, calcium carbonate, fibrous glass, asbestos, components of jet fuel, fire retardants, dioxins and silicates were components of WTC-PM.[13,14] Therefore, our findings in the WTC-exposed FDNY cohort fit into a larger set of studies demonstrating the association of lipids, inflammation, and pulmonary injury and repair after toxin exposure.[1–3]

The Fire Department of New York (FDNY) rescue/recovery workers exposed to WTC-PM have developed obstructive airways disease (OAD).[15–19] The role of classic cardiovascular risk factors in the development of pulmonary disease has been a topic of considerable interest.[19–22] In a WTC-exposed case-cohort study, pulmonary artery to aorta diameter (PA/A) was also associated with early serum biomarkers of vascular disease and predictive of lung disease known as WTC-lung injury (WTC-LI).[23] Metabolic syndrome (MetSyn) phenotypic characteristics also predicted WTC-LI and airway hyperreactivity (AHR).[19,21,22,24–28]

Recently, we focused on discovering metabolic and vascular disease-associated bioactive pathways associated with WTC-LI. Through the use of high-throughput omics technologies, our group has assessed the metabolome of WTC-LI patients.[27] We explored methods to identify a metabolic signature unique to WTC-LI. Automated machine learning techniques exceled in their ability to address potential multicollinearity, and their robustness to false positive and negative discoveries compared to traditional analyses that are based on significance testing. We identified several bioactive classes of lipid and amino acid metabolites.[27,29]

The objective of this study was to develop and validate a multivariate predictive model of WTC-LI by integrating the metabolome with clinical, cytokine, chemokine, and environmental data to improve early identification of disease compared to single-platform models. This process is key to identification of significant biologically active pathways. In this investigation, we have implemented a machine learning approach to first identify and then validate analytes, such as Pigment epithelium-derived factor (PEDF), and their associated pathways that most accurately classify future WTC-LI. While PEDF has been identified as a biomarker of OAD, we have now identified PEDF as a novel, predictive biomarker of the negative health effects of PM exposure.[30]

## Methods

### Ethics statement

Subjects provided written consent to research including biomarker analysis at enrollment (Institutional Review Board approved protocols at Montefiore Medical Center (#07-09-320) and New York University (#16–01412)).

### Study design

**Fig 1:**
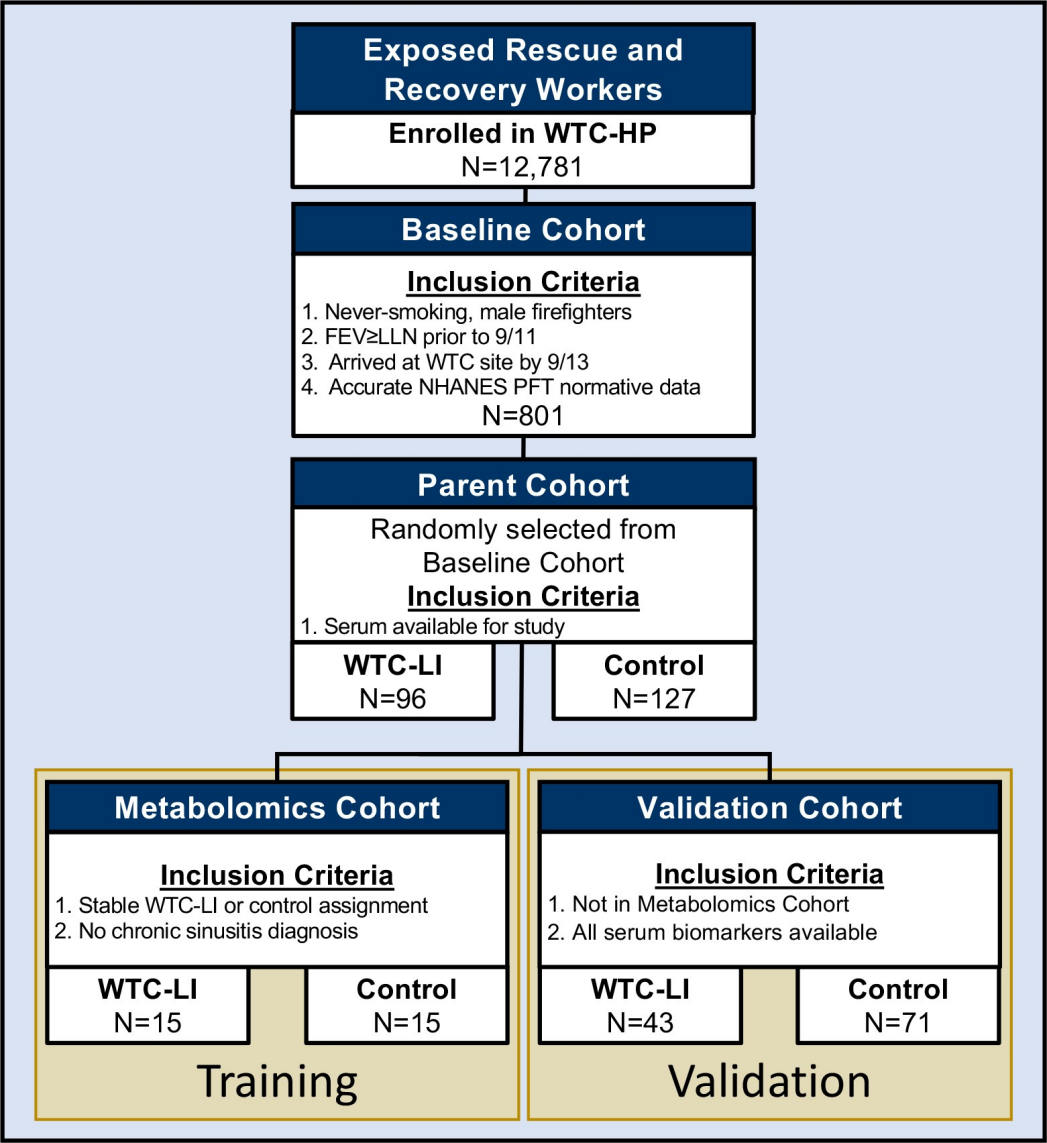
Study Design. From a parent cohort of WTC-LI cases (n = 96) and controls (n = 127), a metabolomics subcohort (n = 15/group) and a validation cohort (n = 114) were drawn as described.

The baseline cohort (N = 801) was obtained from symptomatic subjects referred for subspecialty pulmonary examination (SPE) between 10/1/2001 and 3/10/2008, and underwent pulmonary function testing as previously described.[19,31,32] The ***parent cohort*** consisted of subjects who were male never-smokers with normal pre-9/11/2001 (9/11) lung function, reliable NHANES-predicted FEV_1_, and pulmonary function tests available within 200 days after 9/11, with WTC-LI (n = 96; defined as FEV_1, %Pred_< the lower limit of normal (LLN) at SPE) and randomly selected controls (n = 127) selected as previously described, with the additional criterion of having serum available for analysis.[19,31,32] Subjects (n = 15/group) in the ***metabolomics cohort*** were chosen from the parent cohort for untargeted metabolome assessment if they maintained stable case assignment as previously described, **Fig 1**.[27] This cohort functions as a training set.

The clinical biomarkers and serum cytokines and chemokines identified in the metabolomics cohort were fully available for validation in (n = 43/96) cases of WTC-LI and (n = 71/127) controls that did not overlap with the metabolomics cohort. For clarity, we will refer to this subset of the parent cohort as the ***validation cohort*.** [31] Note that the metabolomics and validation cohorts are disjoint subsets of the parent cohort by design, **Fig 1**.

**Demographics** and clinical data were obtained from the WTC-Health Program (WTC-HP). Exposure intensity is categorized as per the FDNY-WTC Exposure Intensity Index and is based on first arrival time at the WTC site, as described.[15,33,34] Specifically, subjects are considered highly exposed if they arrived the morning of 9/11, intermediate exposure if they arrived the afternoon of 9/11, and low exposure if they arrived on or after September 12, 2001.[28]

### Analytical methods of high throughput OMICs platforms

#### Biomarkers

Serum collected within 200 days after 9/11 was processed and stored as previously described.[19,23,31,32,35] Serum was thawed once and assayed on the following commercially available multiplexed kits (Millipore and R&D) according to manufacturer’s instructions on a Luminex 200IS (Luminex Corporation, TX): cardiovascular (HCVD1-67AK, Millipore), neurodegenerative (HNDG2-36K, Millipore), metabolic (HMH-34K, Millipore), 39-plex (MPXHCYTO-60K, Millipore), soluble receptors (HSCR-32K, Millipore), TIMP (R&D Systems), and Apolipoprotein (APO-62K, Millipore).[19,20,31,36] Samples were processed in approximately 2:1 ratio of controls to cases to avoid batch bias, and analyzed with MasterPlex QT (Version: 1.2; MiraiBio). Serum was quantified in the parent cohort.

#### Metabolomics

Serum aliquots were at -80°C until metabolite quantification. Compounds were matched to corresponding library entries of retention index, mass, and spectral data as previously described.[37–40] Qualified metabolites were detected in 80% or more of subjects per group with a relative standard deviation of 15% or greater.[41] In qualified metabolites, missing data was imputed by using the minimum observed value of each compound, as previously described.[27]

#### Analysis pipeline

Global metabolomic profiling was performed on the metabolomics cohort (n = 15/group). Curated data of the qualified profile (n = 580 metabolites) incorporated with serum analytes quantified via Luminex (n = 106 cytokines, chemokines) and clinical biomarkers (n = 5) into random forests (RF) models consisting of 5,000–500,000 trees in increments of 5,000 trees to determine the minimal amount of trees required to achieve rank stability, defined as zero pairwise differences in prospective refined profile membership among 10 replicate random forests (randomForest 4.6.14, R-Project) similar to previously described, **S1A Fig**.[27] Variables ranking within the top 5% of mean decrease accuracy scores were included in the refined profile. Of the 10 replicate models, we report the mean decrease accuracy of the model with the lowest out-of-bag classification error rate. To account for the potential confounding effects of multicollinearity on classical permutation importance, we measured the permutation importance of each variable conditioned on all other variables in this random forest (i.e. the Conditional Permutation Importance) using a version of the R package permimp 1.0.1 that was customized in-house to support parallel processing.[42–45] To assess the classification accuracy of the refined profile as the out-of-bag error rate, a second iteration of RF was run. The number of trees ranged from 500–15,000 in increments of 500; 10 replicate forests were grown at each increment.[46] Furthermore, we measured variable importance in forests trained using only the refined profile to gain insight into individual biomarker contribution in a lower-dimensional setting. For all random forests, at each node, the square root of the number of total model variables were sampled with replacement. The R packages doParallel 1.0.16, doRNG 1.8.2, and foreach 1.5.1 assisted with parallel processing.

Principal component analysis (PCA) (SPSS 23, IBM) of the correlation matrix of mean-centered, normalized attributes was employed to visualize potential mechanistic relationships between metabolites and other data. The number of components retained was determined based on analysis of the scree plot. Unsupervised two-way hierarchical clustering was performed on the refined profile’s data matrix using Spearman correlation and average linkage (Matlab R2018a). Linkage thresholds of 0.78 and 1.10 were used to define clusters of metabolites and subjects, respectively.

#### Validation

Cytokines, chemokines, and clinical biomarkers identified in the refined profile of the metabolomics cohort were fully available for validation of a multivariate predictive model of WTC-LI in the non-overlapping parent cohort via binary logistic regression. There are several ways to build predictive models of disease based on a subset of important features. These include support vector machines which allow flexible modelling of nonlinear effects and interactions among biomarkers. We chose binary logistic regression to construct a linear combinator of identified biomarkers for differentiating cases from controls that would be interpretable.[31] Least absolute shrinkage and selection operator (LASSO) was used to identify an optimal subset of serum analytes in a binary logistic regression (R package glmnet 4.1).[47,48] Additional variables were included based on high mean decrease accuracy in the RF of the metabolomics cohort. Variables were dichotomized using Youden’s index. Multicollinearity was assessed on continuous variables via Pearson correlation, and, where significant (p<0.05), handled via generation of composite variables post-dichotomization. Performance of logistic regressions was assessed via AUC_ROC_ (R package pROC 1.16.2). We have opted to construct receiver-operator characteristic (ROC) curves and report AUC_ROC_ as the main criteria of the model because ROC curves and AUC_ROC_ are appropriate in the setting of balanced classes. The final model underwent 5-fold cross-validation to assess generalizability (R package boot 1.3.25).[49] All logistic regressions were confounder-adjusted for age on 9/11, BMI at SPE, exposure, and Pre-9/11 FEV_1, %Pred_.

### Statistics, database management and multivariate model development

SPSS 23 (IBM) was utilized for data storage and handling. Data analysis was performed in R (3.6.0 and 4.0.3, R-Project). Continuous and ordinal variables were expressed as median and inter-quartile range. Wilcox test was used to compare continuous and ordinal data. For categorical data, count and proportions were used to summarize and Pearson-χ^2^ was used for comparison. For all tests, p<0.05 was considered significant. Correlations were calculated using the R package Hmisc 4.4.2. SPSS file formats were read and written in R haven 2.3.1. Additionally, the R package matrixStats 0.57.0 was used to perform some row- and column-wise computations. Finally, ggplot2 3.3.2 was used for tune result visualizations.

### Computing resources

Big Purple (https://hpcmed.org/resources) was used for computing and includes Cray CS500, Skylake 6148, 20-core, 2.4GHz, 150W processors, 32GB DDR4-2666 DIMMs (DUAL RANKED), 384 GB/node, 9.6 GB/core, 2TB SATA disk, 2TB NVMe SSD and an EDR 100Gb/s Infiniband network interface for MPI and Data traffic. Additional, hardware details can be found at (https://hpcmed.org/resources/bigpurple/hardware).

## Results

### Demographics

The metabolomics cohort has been previously comprehensively described in terms of clinically available lipids, leukocyte differentials, and metabolic biomarkers.[27] The parent cohort has also been previously described in terms of its available clinical characteristics.[20] In the present study, we did not consider differential expression of serum cytokines and chemokines in the parent cohort. We additionally provide clinical characteristics and serum cytokines and chemokines of the metabolomics and validation cohorts used in this paper, **Table 1**.

**Table 1:** Clinical measures for primary endpoint.

		WTC-LI (N = 43)	Control (N = 71)	WTC-LI (N = 15)	Control (N = 15)
**Age on 9/11 (y)**		**41** (36–46)	**41** (37–44)	**39** (37–46)	**42** (38–46)
**Race**	**Caucasian**	**39** (91%)	**70** (99%)	**15** (100%)	**15** (100%)
	**African American**	**4** (9%)	**1** (1%)	**0** (0%)	**0** (0%)
**BMI at SPE (kg/m**^**2**^**)**		**29.41** (27.47–34.20)	**29.74** (26.90–31.57)	**30.28** (27.80–31.42)	**25.66** (24.40–27.98)
**BMI at WTC-HP (kg/m**^**2**^**)**		**28.97** (27.26–32.45)	**28.59** (26.97–30.85)	**29.29** (25.82–31.24)	**25.84** (25.10–27.37)
**Pre-9/11 FEV**_**1, %Pred**_		**95** (83–105)	**104** (94–113)	**85** (83–90)	**97** (92–105)
**SBP (mmHg)**		**120** (110–132)	**120** (110–128)	**119** (108–129)	**110** (100–112)
**Exposure**	**Low**	**7** (16%)	**8** (11%)	**4** (27%)	**1** (7%)
	**Intermediate**	**24** (56%)	**49** (69%)	**8** (53%)	**11** (73%)
	**High**	**12** (28%)	**14** (20%)	**3** (20%)	**3** (20%)
**PEDF (pg/cL)**		**1.70** (1.12–3.86)	**4.03** (1.41–5.03)	**1.14** (0.71–4.24)	**5.29** (4.39–5.73)
**MDC**		**1517.59** (1254.37–1988.01)	**1428.08** (1074.94–1837.64)	**1899.30** (1649.77–2312.16)	**1334.72** (892.12–1395.58)
**MIP-4 (ng/mL)**		**155.65** (105.07–201.04)	**198.08** (123.85–1036.74)	**137.74** (106.58–283.08)	**1207.36** (194.90–1674.58)
**GRO**		**707.87** (460.34–1094.75)	**708.49** (567.38–929.57)	**811.81** (671.70–1143.64)	**485.20** (412.83–615.32)
**MCP-1**		**543.18** (366.42–765.20)	**544.07** (403.57–654.20)	**589.41** (495.64–1087.35)	**398.23** (314.11–493.11)
**sIL-2Rα**		**555.01** (462.04–741.69)	**535.63** (390.08–787.68)	**573.39** (534.55–736.32)	**419.91** (273.28–580.45)
**Amylin**		**53.45** (35.40–67.48)	**58.39** (46.37–147.64)	**53.45** (46.20–55.12)	**63.25** (55.12–153.36)
**sCD40L**		**9032.94** (5223.64–16242.28)	**8306.12** (4893.05–15116.64)	**15834.16** (8255.89–28494.18)	**5070.92** (712.82–9163.09)
**MMP-1**		**402.79** (141.08–767.15)	**861.41** (337.84–1441.27)	**130.70** (75.64–633.73)	**647.26** (286.43–1368.21)
**sVEGFR1**		**457.86** (343.76–626.35)	**457.86** (343.76–627.62)	**480.31** (249.97–749.74)	**377.41** (292.12–420.08)
**EGF**		**64.14** (29.11–125.71)	**64.88** (43.50–132.55)	**117.37** (33.49–246.65)	**47.23** (23.42–74.38)
**Leptin**		**7756.10** (4638.00–13557.20)	**6428.99** (4021.21–10479.97)	**8377.03** (5583.98–18744.24)	**4344.83** (1964.47–6250.28)
**Apo All (μg/mL)**		**1818.81** (632.93–2479.78)	**780.70** (418.96–1594.96)	**913.40** (319.21–4298.69)	**673.30** (538.10–1554.70)
**MMP-13**		**61.89** (9.98–117.47)	**74.65** (8.94–133.06)	**12.65** (8.93–101.87)	**113.16** (2.00–177.31)

Values are in Median (IQR) or N (%) as indicated; p calculated by Wilcox test or Chi-Square as appropriate; Apo AII available for 14 controls in metabolomics cohort.

Analytes are pg/mL unless otherwise stated. Apo AII is shown in units μg/mL for readability but analyzed in ng/mL.

**In the metabolomics cohort**, cases of WTC-LI had significantly elevated SBP, BMI at WTC-HP and SPE, MDC, GRO, MCP-1, sIL-2Rα, sCD40L, EGF, and Leptin, and significantly decreased Amylin compared to controls. Additionally, cases of WTC-LI and controls were not significantly different in Apo-AII.

**In the validation cohort**, cases of WTC-LI had significantly elevated Apo AII compared to controls; however, cases of WTC-LI and controls were not significantly different in BMI at WTC-HP or SPE, systolic blood pressure (SBP), MDC, GRO, MCP-1, sIL-2Rα, Amylin, sCD40L, EGF, or Leptin. Finally, there were no observed differences in clinical biomarkers or serum analytes between controls in the validation cohort and those in the parent cohort, nor were there differences in clinical biomarkers or serum cytokines and chemokines between cases of WTC-LI in parent and validation cohorts.

**In both the metabolomics and validation cohorts**, cases of WTC-LI had significantly decreased pre-9/11 FEV_1, %Pred_, PEDF, MIP-4, and MMP-1 compared to controls; however, there were no significant differences in age on 9/11, race, exposure, sVEGFR1, and MMP-13 between cases of WTC-LI and controls in either cohort.

Finally, controls in the validation cohort significantly differed from controls in the metabolomics cohort in BMI at WTC-HP and SPE, SBP, PEDF, MDC, GRO, MCP-1, sIL-2Rα, sCD40L, and Leptin; however, cases of WTC-LI in the validation cohort did not differ from cases of WTC-LI in the metabolomics cohort in clinical biomarkers or serum cytokines and chemokines. Furthermore, relative expression of cases of WTC-LI compared to controls were preserved for these biomarkers across both cohorts, with the exceptions of BMI at SPE, SBP, GRO, and MCP-1, which were elevated in cases of WTC-LI compared to controls in the metabolomics cohort, but had a fold change of approximately 1 in cases of WTC-LI compared to controls in the validation cohort.

### Metabolomics

**Fig 2:**
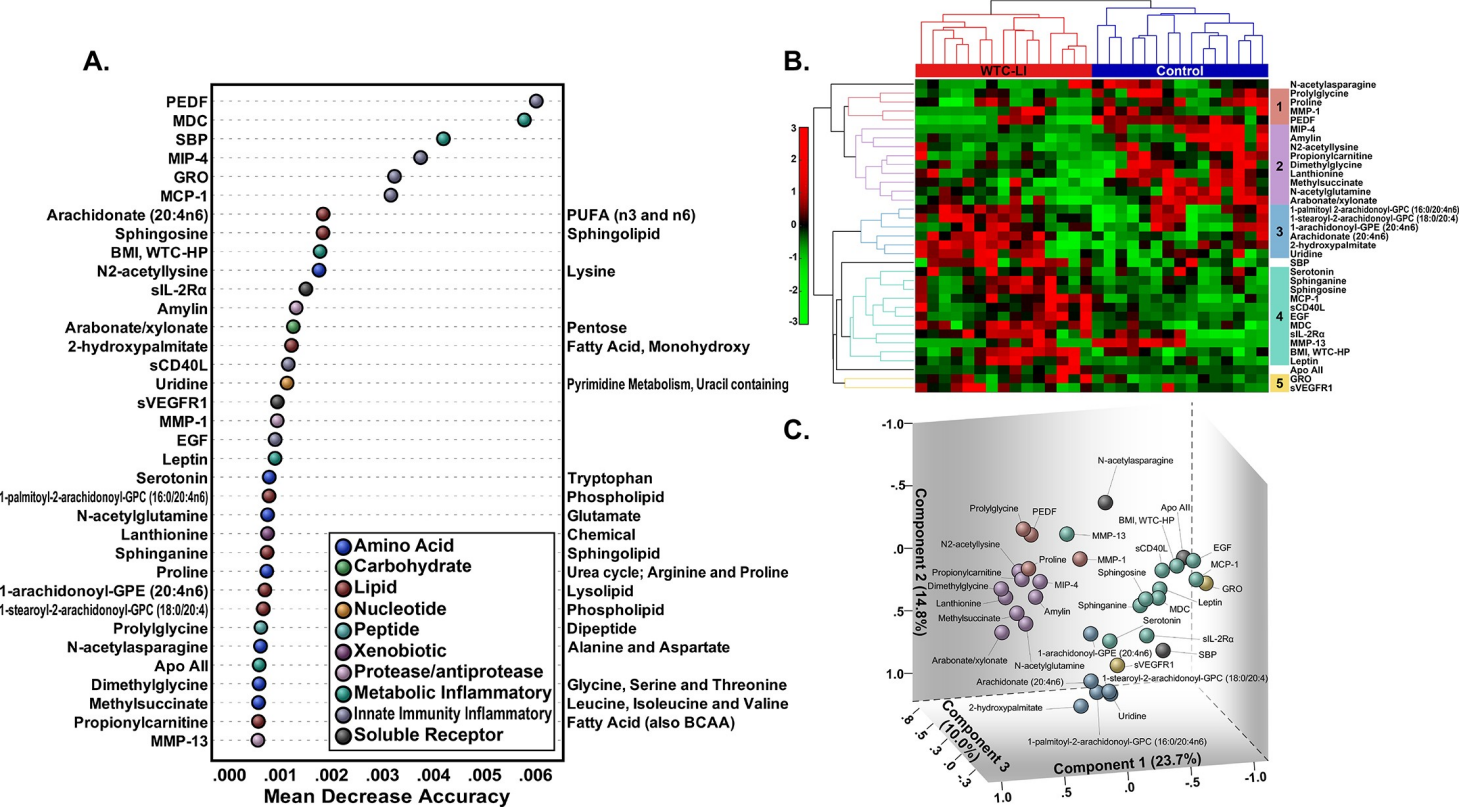
Random Forests Variable Importance. **A.** Mean decrease accuracy was used to determine and rank the top 5% of important metabolites, cytokines, chemokines, and clinical biomarkers. **B. Agglomerative, Hierarchical Clustering** identified 5 clusters of variables in the refined profile with similar patterns of expression in the metabolomics subcohort. **C. PCA Loading Weights Plot** visualizes clusters of variables based on intervariable correlations and provides an alternative-but-similar view of variable relationships in the metabolomics subcohort. Points are colored according to cluster membership.

We have previously characterized 765 detected and 580 qualified metabolites.[27] We included the qualified metabolites in RF with serum, clinical, and environmental biomarkers to assess the most discriminative variables as those with a mean decrease accuracy score within the top 5% of scores. The tuning process determined the minimum number of trees (n = 345,000) that resulted in 0 average pairwise unique elements among prospective refined profiles of 10 replicate random forests, **S1A Fig**. This yielded a refined profile of 19 metabolites (largely sphingolipids, phospholipids, fatty acids, and amino acids), 14 serum biomarkers (including protease/antiprotease, metabolic inflammatory, innate immunity inflammatory, and soluble receptor biomarkers), and 2 clinical biomarkers (BMI at WTC-HP entry and SBP), **Fig 2A**. Membership in the refined profile was identical using the variable importance ranking produced by conditional permutation importance, **S1B Fig**. A second RF trained on only the refined profile achieved a 0% estimated out-of-bag error rate with 1,500 trees, **S2A Fig**. Furthermore, an additional analysis of variable importance was performed using the smallest forest (1,500 trees) to achieve the minimal error rate. Variable importance ranking derived from both classical and conditional permutation importance were similar to each other (Spearman’s rank correlation of 0.942, p<0.001) and to the variable importance ranking derived using the qualified profile (Spearman’s rank correlations of 0.812, p<0.001 and 0.770, p<0.001, respectively), **S2B and S2C Fig**.

Clustering of the data matrix of the refined profile identified 5 clusters (C1-5) reflective of potential mechanistic relations, **Fig 2B**. C1—elevated in controls—contained vascular biomarkers, as well as amino acids and peptides. In C2, amino acids predominated and were decreased in WTC-LI. Fatty acids primarily comprised C3, many of which were elevated in cases of WTC-LI, and in controls. Variables in C4 were also generally elevated in WTC-LI, and included vascular biomarkers (sphingolipids, SBP). Finally, C5 contained sVEGFR1 and GRO, which were elevated in cases of WTC-LI.

PCA of the refined metabolite profile captured 73.3% of variance in the 7 components retained based on examination of the scree plot. The PCA loading weights plot provides an alternative, low-dimensional view of the variables in C1-5, and the clustering patterns observed in the loading weights plot are reflective of the structure of C1-5.

### Validation of serum and clinical biomarkers in WTC-LI vs. controls

RF analysis uncovered several previously unidentified serum biomarkers in the metabolomics cohort. Using binary logistic regression, we aimed to validate these serum biomarkers in the validation cohort.

### Biomarkers identified by integrated MultiOMICs approach

To analyze the relationship between all cytokines, chemokines, and clinical biomarkers identified in RF as continuous variables and potential confounders, we transformed exposure into dummy variables and determined an optimal model using LASSO set to maximize 5-fold cross-validated AUC_ROC_. Metabolites were not fully available in the validation cohort and so were not included in the use of LASSO. MIP-4, MMP-1, Apo AII, MDC, Amylin, Pre-9/11 FEV_1, %Pred_, and intermediate exposure remained in the model with AUC_ROC_ (Standard Error) of 0.728(0.066), **Table 2** and **S3 Fig**.

**Table 2:** LASSO regression.

		Coefficient
**MIP-4**		-1.427 e-04
**MMP-1**		-3.168 e-04
**Apo AII**		1.498 e-08
**MDC**		1.659 e-04
**Amylin**		-9.477 e-04
**Pre-9/11 FEV**_**1, %Pred**_		-3.010 e-02
**Exposure**	**Intermediate**	-8.066 e-02

Analyte units: MIP-4 and Apo AII ng/mL; MMP-1 pg/mL.

To handle potential multicollinearity, we then included other variables that were important predictors in the metabolomics cohort, but were significantly correlated (p<0.05) with other variables in the initial regression, **Fig 2**. Here, we considered the top 6 variables by mean decrease accuracy in the refined profile—there was appreciable drop in mean decrease accuracy after these variables.

Univariate regression analysis was conducted to characterize the relationship between WTC-LI and the analytes of interest identified via LASSO or the refined biomarker profile—PEDF, MDC, MCP-1, MIP-4, GRO, SBP, MMP-1, and Apo AII, **Table 3**.

**Table 3:** Univariate and composite models.

	Univariate OR (95% CI)	Univariate p	AUC_ROC_ (95% CI)
**GRO**	**1.000** (0.999–1.001)	0.495	**0.711** (0.608–0.815)
**SBP (mmHg)**	**0.991** (0.957–1.026)	0.612	**0.719** (0.616–0.822)
**MCP-1**	**1.000** (0.998–1.002)	0.776	**0.710** (0.607–0.812)
**MDC**	**1.001** (1.000–1.001)	0.100	**0.717** (0.615–0.819)
**Apo AII (ng/mL)**	**1.000** (1.000–1.000)	0.077	**0.741** (0.644–0.837)
**MMP-1**	**0.999** (0.998–1.000)	**0.012**	**0.755** (0.661–0.849)
**MIP-4 (ng/mL)**	**0.998** (0.996–0.999)	**0.017**	**0.783** (0.697–0.869)
**PEDF (pg/cL)**	**0.824** (0.665–0.978)	0.058	**0.743** (0.647–0.838)
**Apo AII > 1794.22 (μg/mL)**	**8.044** (3.086–22.931)	**<0.001**	**0.812** (0.729–0.895)
**MMP-1 > 832.48**	**0.173** (0.062–0.440)	**<0.001**	**0.799** (0.713–0.884)
**SBP > 127.5 (mmHg)**	**1.280** (0.460–3.539)	0.633	**0.714** (0.610–0.817)
**GRO > 580.28, MCP-1 > 279.07, and MDC < 1756.99**	**0.327** (0.127–0.787)	**0.015**	**0.760** (0.665–0.854)
**PEDF < 3.94 (pg/cL) and MIP-4 < 368.70 (ng/mL)**	**5.252** (2.150–13.900)	**<0.001**	**0.797** (0.712–0.882)

Analytes are pg/mL unless otherwise stated. All models were adjusted for the potential confounders age on 9/11, BMI at SPE, pre-9/11 FEV_1, %Pred_, and exposure.

This process provides insight into the contribution and performance of single biomarkers as measured by OR and AUC_ROC_, **Table 3**. MMP-1 and MIP-4 were significant in univariate, confounder-adjusted models.

In building the final model, we optimized biomarker cutpoints via Youden’s Index. To account for significant correlations observed in **Fig 2B**, we created composite variables. The relationship between these variables and WTC-LI was analyzed in confounder-adjusted binary logistic regressions, which are summarized along with AUC_ROC_ as a performance measure, **Table 3**. Apo AII>1794.22μg/mL, MMP-1>832.48pg/mL, (PEDF<3.94pg/cL and MIP-4<368.70ng/mL), and (GRO>580.28pg/mL, MCP-1>279.07pg/mL, and MDC<1756.99pg/mL) were significant in univariate, confounder-adjusted models, while SBP>127.5mmHg was not significant.

**Fig 3:**
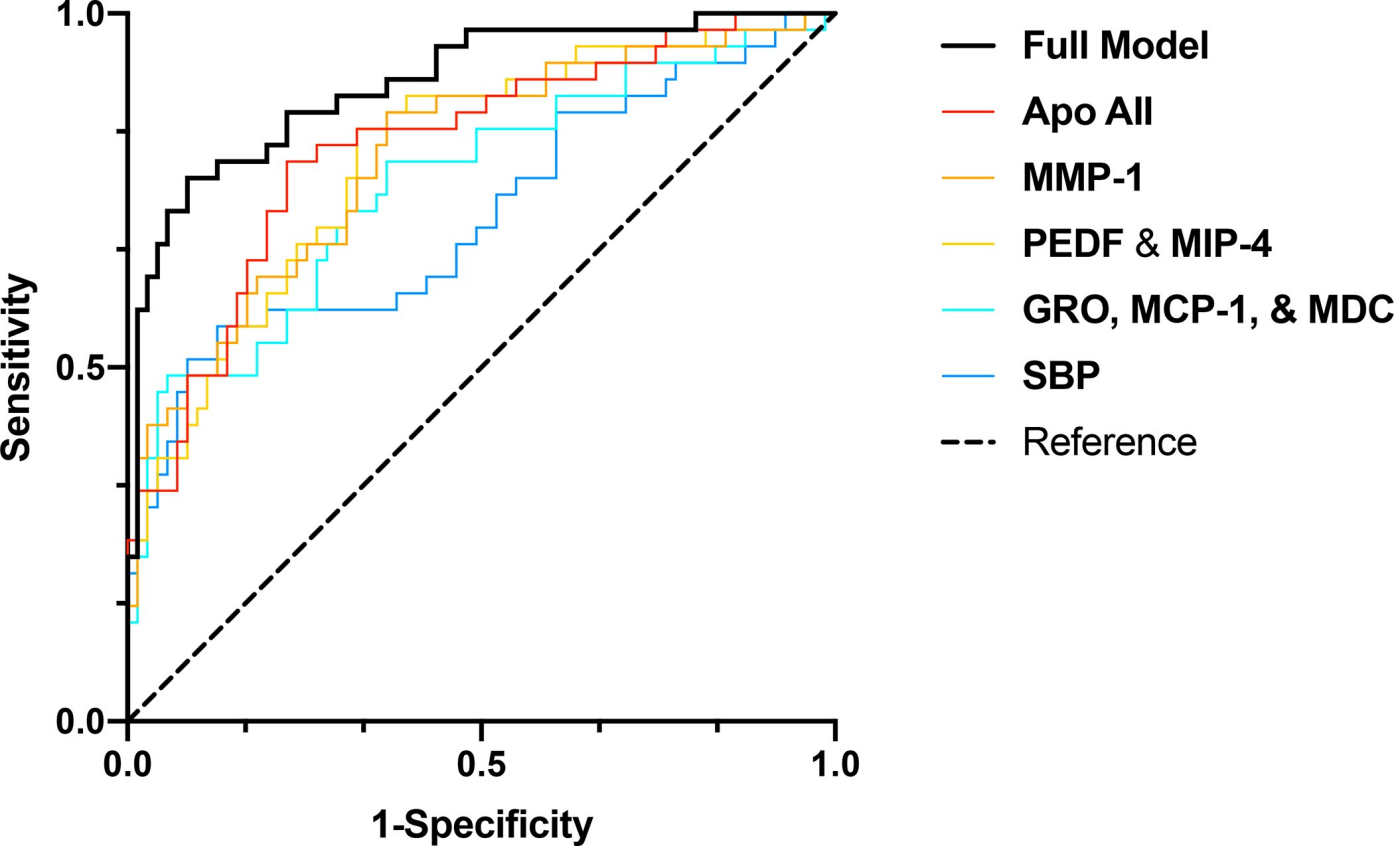
Predictive performance of Final Model. **ROC**_**AUC**_ of the final, confounder-adjusted, multivariate binary logistic regression shows its predictive performance as well as that of its constituents when entered separately in confounder-adjusted binary logistic regressions.

The variables initially identified via LASSO were then transformed to dichotomous variables using Youden’s index. These variables, along with dichotomous and composite variables corresponding to those with the highest mean decrease accuracy score within the refined profile, were included in a final, confounder-adjusted, binary logistic regression, **Table 4** and **Fig 3**.

**Table 4:** Final multivariate model.

		OR (95% CI)	p
**PEDF < 3.94 (pg/cL) and MIP-4 < 368.70 (ng/mL)**		**8.874** (2.112–47.363)	**0.005**
**SBP > 127.5 (mmHg)**		**2.122** (0.567–8.481)	0.270
**Apo AII > 1794.22 (μg/mL)**		**15.445** (4.566–66.932)	**<0.001**
**MMP-1 > 832.48**		**0.375** (0.083–1.569)	0.185
**GRO > 580.28, MCP-1 > 279.07, and MDC < 1756.99**		**0.299** (0.085–0.951)	**0.047**
**Age on 9/11 (y)**		**1.010** (0.922–1.104)	0.823
**BMI at SPE (kg/m**^**2**^**)**		**0.936** (0.828–1.055)	0.268
**Pre-9/11 FEV**_**1, %Pred**_		**0.946** (0.902–0.983)	**0.010**
**Exposure**	**Low**	**Reference**	
	**Intermediate**	**0.235** (0.042–1.151)	0.082
	**High**	**0.551** (0.076–3.689)	0.540

Analytes are pg/mL unless otherwise stated.

PEDF<3.94pg/cL and MIP-4<368.70ng/mL, (GRO>580.28pg/mL, MCP-1>279.07pg/mL, MDC<1756.99pg/mL), and Apo AII>1794.22μg/mL, were significant in this model, which achieved an **AUC**_**ROC**_ **of 0.902** (95% CI 0.842–0.961), **Fig 3**. Furthermore, we validated the final multivariate model using 5-fold cross-validation. The estimated bias-corrected prediction error was 0.160.

## Discussion

Lung injury is heterogeneous in cause, process, and outcome. The biomarkers found to be important to the development of WTC-LI similarly are heterogenous.[19,20,22–28,31,32,34–36,50–54] The integration of high-throughput platforms with classical clinical measurements, serum cytokines, and chemokines represent a promising avenue to characterization of disease and identification of therapeutic targets for lung injury; however, approaching such a high-dimensional dataset may be restrictively complex. In this paper, we presented an automated data pruning method and successfully integrated metabolomics data with a longitudinal dataset of serum cytokines and chemokines, and other clinical measures in 9/11 FDNY rescue and recovery workers exposed to WTC-PM.

We discovered several novel biomarkers of WTC-LI—including PEDF—in a cohort with untargeted metabolomics, and analyzed biomarker relationships in a hypothesis-generating fashion to identify potential cellular signaling cascades. We then validated the predictive ability of the cytokines, chemokines and clinical biomarkers in a broader cohort.

**PEDF**—the most important variable in RF of the metabolomics subcohort and one of the most predictive in univariate, confounder-adjusted binary logistic regressions—is a pleiotropic glycoprotein that belongs to the serpin superfamily of serine protease inhibitors.[55] PEDF is a multifunctional protein with important roles in regulation of inflammation and angiogenesis, and has been implicated in several lung injury patterns. It is produced by various cell types, including endothelial cells. In pulmonary fibrosis, PEDF is an angiostatic factor.[56] Furthermore, PEDF expression contributes to modulation of the inflammatory and angiogenic phenotype of the lung endothelium, which is key to several conditions such as pulmonary hypertension.[57] This non-inhibitory protein plays critical roles in many physiological and pathological processes by acting through multiple high affinity ligands and cell receptors.[55,58] PEDF is notably involved in organogenesis and the homeostatic maintenance of adult tissues/organs. The mRNA that encodes PEDF (*SERPINF1* mRNA) is expressed in most tissues/organs. In addition to regulating fibrosis in the lung, PEDF regulates angiogenesis in the lung, pancreas, kidney, and eye, as well as lipid metabolism in the liver. PEDF also plays a role in bone cell differentiation. Deficiencies or defects of PEDF protein expression can lead to abnormal organ development and are closely associated with the progression of angiogenic diseases.[58] Because of PEDF’s abundance and variety of functions in the body, PM-associated PEDF alterations could be a mediator of systemic effects secondary to oropharyngeal aspiration of or inhalational exposure to PM. Clinical studies have shown that PEDF is correlated with idiopathic pulmonary fibrosis (IPF), COPD, lung cancer, MetSyn, and diabetes.[58,59] Specifically, elevated PEDF levels are involved in the pathogenesis of COPD and IPF.[30,56] In a study by Li et al., PEDF expression levels were upregulated in both cigarette smoke extract-stimulated epithelial cells and cigarette smoke-exposed rat lung.[30] Additionally, a significant, negative correlation between PEDF levels and lung function was shown, with plasma PEDF levels in COPD patients significantly higher than those in both the healthy nonsmoking and smoking subjects. These results indicate the elevation of PEDF plays a role in the pathogenesis of COPD by mediating inflammatory signaling processes.[30]

Reduced PEDF expression facilitates the progression of lung cancer due to the loss of PEDF-related suppression of tumor growth and motility.[60] PEDF is an adipocyte-secreted protein that acts as a pro-inflammatory factor by activating inflammatory signaling in several cell types. Therefore, PEDF contributes to the onset and maintenance of chronic inflammation in obesity and obesity-induced insulin resistance, and related complications such as MetSyn and type 2 diabetes mellitus.[61,62] Serum levels of PEDF are higher in patients with MetSyn and type 2 diabetes.[62,63] PEDF expression is elevated in proportion to the accumulation of the number of components of MetSyn in the general population. Nakamura et al. demonstrated that waist circumference, triglycerides, and creatinine were significant independent determinants of serum PEDF levels in diabetics; [63] however, strong, well-known risk factors such as age, blood pressure, and smoking were not related to PEDF.[63] PEDF has been shown to have neurotrophic and neuroprotective effects, and has been implicated in the pathogenesis of Alzheimer’s Disease.[64]

Additionally **MMP-1**, a protease which has been implicated in lung fibrosis, interstitial pneumonia, and cancer, was found to be important in our model.[65,66] Proteases have a prominent role in cancer, coronary disease, and OAD.[67–71] Increased protease activity is a component of many diseases, including cigarette-induced chronic lung disease and other causes of accelerated lung function decline.[67–70] MMPs’ central role in lung remodeling and pathogenesis of OAD has been of particular interest. MMPs are a family of Zn^2+^-dependent proteases that can catabolize and degrade the extracellular matrix. Levels of MMPs are affected by environmental factors such as hypoxia, inflammation, and oxidative stress; MMPs as biomarkers of lung disease severity and prognostic indicators have been investigated in several studies.[67,72–74]

### A metabolic hotspot

In C4, sphingolipids (sphinganine, sphingosine) displayed similar expression as other regulators of vascular proliferation and metabolic indicators (BMI, Leptin) and several immune-cell signaling molecules (IL-2Rα, sCD40L, **MCP-1, and MDC**). Biomarkers of inflammation such as **MDC** and of MetSyn, were observed in serum drawn within 6 months of WTC exposure, and predicted the post-9/11 loss in FEV_1_ in this cohort of WTC-exposed FDNY firefighters.[19,31] The proximity of drivers of vascular proliferation to markers of metabolic dysregulation in this cluster is interesting in the context of the potential interaction between angiogenesis and adipogenesis.[75] Furthermore, the presence of immune-cell signaling molecules in this cluster could be reflective of a phenotype of chronic inflammation concomitant with obesity that has been observed in this cohort. Our prior works have found that triglycerides≥150mg/dL and BMI≥30kg/m^2^ impart more risk on development of WTC-LI than smoking or exposure alone. We also know that there is a dose-response with increasing number of MetSyn characteristics and risk of WTC-LI.[19,22,24–28] Many of these mediators are also important in acute lung injury and fibrosis.[76,77]

In contrast to C4, C1 displayed an opposite elevation pattern. Containing PEDF, the decreased expression of C1 further supports a claim of potentially excessive angiogenesis that has been associated with lung diseases, including asthma, as well as PEDF’s previously discussed myriad consequences of dysregulation. Furthermore, in C5, sVEGR1’s moderate elevation in WTC-LI is consistent with this picture.

C2 was the most similar cluster to C1 and contained **Amylin** and **MIP-4**. These biomarkers are metabolically active (Amylin) and mediators of inflammation in adaptive immunity (MIP-4). Therefore, their clustering proximity is of interest in the context of the interaction of metabolic heath, inflammation, and pulmonary health. **MIP-4** is a chemokine and involved in both innate and adaptive immunity. MIP-4 (CCL18) is an early promoter of Treg differentiation and may generate an anti-inflammatory counter-regulatory response.[20,78,79]

Additional biomarkers were identified as being associated with WTC-LI. Similar to MCP-1, MDC, and MIP-4, **GRO** was also found to be important in our WTC-LI model and further revealed immune cell involvement in WTC-LI pathogenesis.[80] GRO—also relevant in acute and chronic lung inflammation such as fibrosis—is part of a chemokine receptor system that mediates neutrophil recruitment.[81–84] In addition, lipids are a diverse group of bioactive compounds that have been implicated in the development of lung disease. Elevated levels of **Apo AII** were associated with pulmonary arterial hypertension in Sickle cell disease, suggesting a role in pulmonary vascular injury.[85] This is in line with our prior observation that dyslipidemia predicts poor outcome after WTC dust exposure.[19,20]

In light of the integrated metabolomics and the added granularity in phenotyping, the findings of our final multivariate binary logistic regression show the strength of early-identification of disease that may predict a non-resolving pathology. In comparison to prior work, the integrated multiplatform model developed here had 6.7% improved performance compared to a metabolomics-only model of disease in this cohort.[27] Furthermore, the final model based on serum and clinical biomarkers identified by the data analysis pipeline in the present study had higher AUC_ROC_ (0.858 vs. 0.902) compared to previous work in a similar cohort.[20] Future investigation will include contemporary assessment of the metabolome, and should also include additional assessment of serum cytokines and chemokines. Such research could determine the degree to which present-day omics displays features predicted by early-disease biomarkers, and the potential these features have as therapeutic targets both proximal to exposure and years later.

### This study has several limitations

The metabolome is only assessed at a single time point, and therefore limits our ability to understand how longitudinal metabolomic variations relate to the development of WTC-LI. Given the size of the metabolomics analysis cohort, we attempted to minimize confounding effects by selection from a homogeneous subject pool; cases of WTC-LI and controls in the metabolomics cohort only differed in BMI, SBP, and HR, but were no different in other metabolic and inflammatory biomarkers. In our metabolomics cohort, we controlled for baseline effects due to BMI variations via percent-predicted-based case definitions.

Finally, the limitations of the present analysis pipeline have been previously described.[27,29] Briefly, any machine learning model is specific to its training data. Given the size of the metabolomics cohort, we have used machine learning methods that avoid overfitting. We cannot support claims of causality from the present analysis, but we can identify potentially important associations and support our findings with relevant literature. While we lack an external validation cohort for the trends observed in metabolomics, we have validated the serum, clinical, and environmental biomarkers via the binary logistic regression model built on the validation cohort. Note that the metabolomics and validation cohorts are subject to the same pre-analytical and analytical biases, and thus our biomarkers have yet to be evaluated in a truly independent test cohort. To address multicollinearity in the high-dimensional integrated dataset of metabolites, clinical, and other serum biomarkers, we assessed variable importance via conditional permutation importance, but found no differences in refined profile membership compared to classical permutation importance. To handle multicollinearity in the final model, we used dichotomous and composite variables. Dichotomization can reduce statistical power. So, we assessed generalizability of our model using 5-fold cross validation.[86]

### Conclusion

Automated data pruning successfully identified a set of maximally discriminative biomarkers in a high-dimensional systems biology dataset and validated these biomarkers in an independent cohort using standard regression techniques. Additionally, we have used pattern recognition methods to visualize associations between maximally predictive metabolites and biomarkers, and potentially elucidate mechanistic relations. PEDF may be an important mediator in WTC-LI and OAD. Future research to discover PEDF’s role in pathogenesis will include targeted molecular imaging and transcriptional quantification to determine PEDF’s activity at relevant tissues and organs.

## Supporting information

S1 Fig **A. Random Forests Hyperparameter Tuning.** Variable rank stability among the top 5% of important variables by mean decrease accuracy was assessed and the minimal number of trees required to achieve stability, defined as no differences among prospective refined profile membership among 10 replicate models, was used. The vertical line intersects the horizontal axis at 345,000 trees. **B. Conditional Permutation Importance.** Using the random forest that derived mean decrease accuracy in Fig 2A, conditional permutation importance was assessed to determine if multicollinearity affected variable ranking.
(TIF)

S2 Fig Tuning process using refined profile. **A.** Mean estimated out-of-bag error rate for 10 replicate forests grown at each increment of forest size, with forest size ranging from 500 to 15,000 trees. **B.** Mean decrease accuracy and **C.** Conditional permutation importance for the smallest forest that achieved the minimum estimated out-of-bag error rate.
(TIF)

S3 Fig LASSO tuning procedure. Tuning process maximized 5-fold cross-validated AUC_ROC_ as a function of log(λ).
(TIF)

S1 Table Overview of Analytes and Representative References. (DOCX)
