# Supplementary figures and images for: PEDF, a pleiotropic WTC-LI biomarker: Machine learning biomarker identification and validation
Source: PLoS Comput Biol. 2021 Jul 21;17(7):e1009144. doi: 10.1371/journal.pcbi.1009144 (PMC8328304; doi:10.1371/journal.pcbi.1009144)

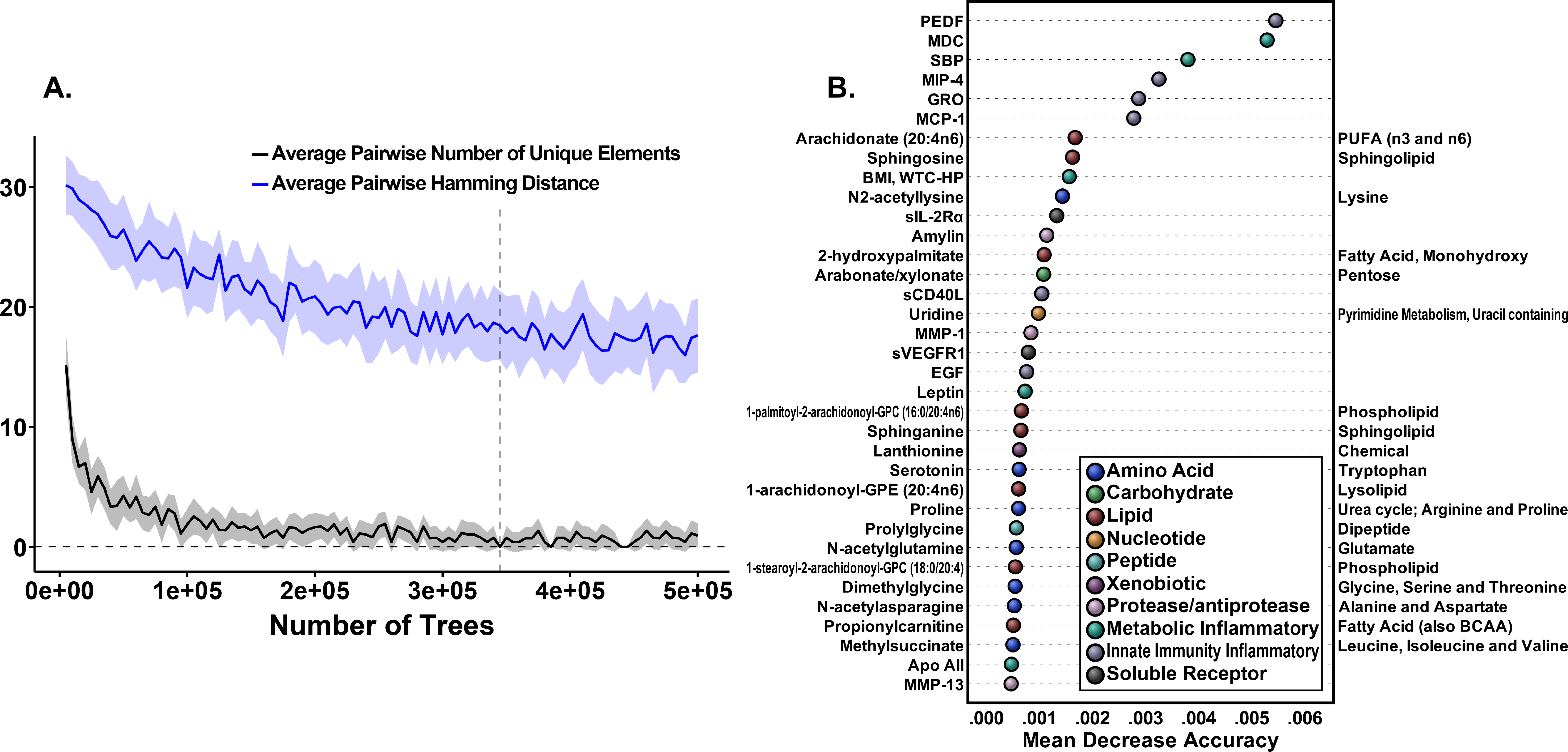

Supplement: S1 Fig — A. Random Forests Hyperparameter Tuning. Variable rank stability among the top 5% of important variables by mean decrease accuracy was assessed and the minimal number of trees required to achieve stability, defined as no differences among prospective refined profile membership among 10 replicate models, was used. The vertical line intersects the horizontal axis at 345,000 trees. B. Conditional Permutation Importance. Using the random forest that derived mean decrease accuracy in Fig 2A, conditional permutation importance was assessed to determine if multicollinearity affected variable ranking. (TIF) [file pcbi.1009144.s001.tif]

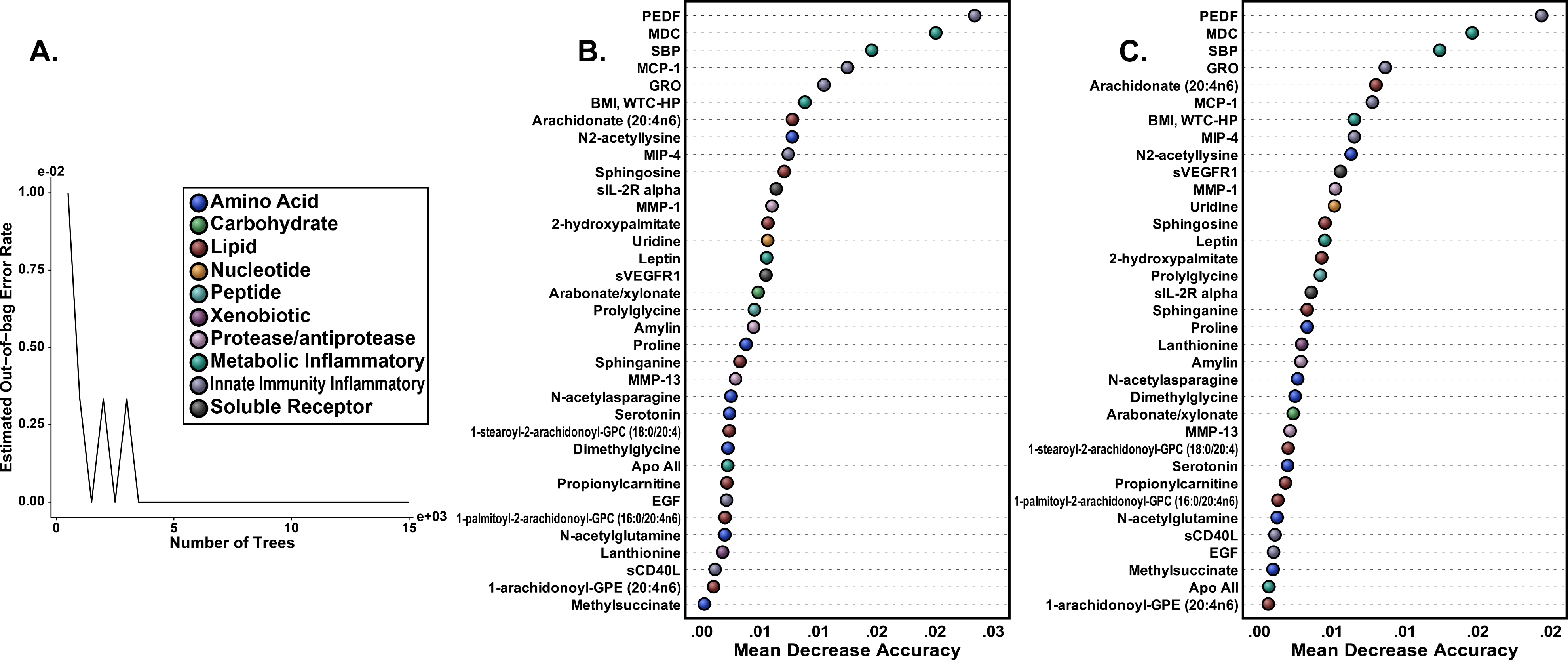

Supplement: S2 Fig — A. Mean estimated out-of-bag error rate for 10 replicate forests grown at each increment of forest size, with forest size ranging from 500 to 15,000 trees. B. Mean decrease accuracy and C. Conditional permutation importance for the smallest forest that achieved the minimum estimated out-of-bag error rate. (TIF) [file pcbi.1009144.s002.tif]

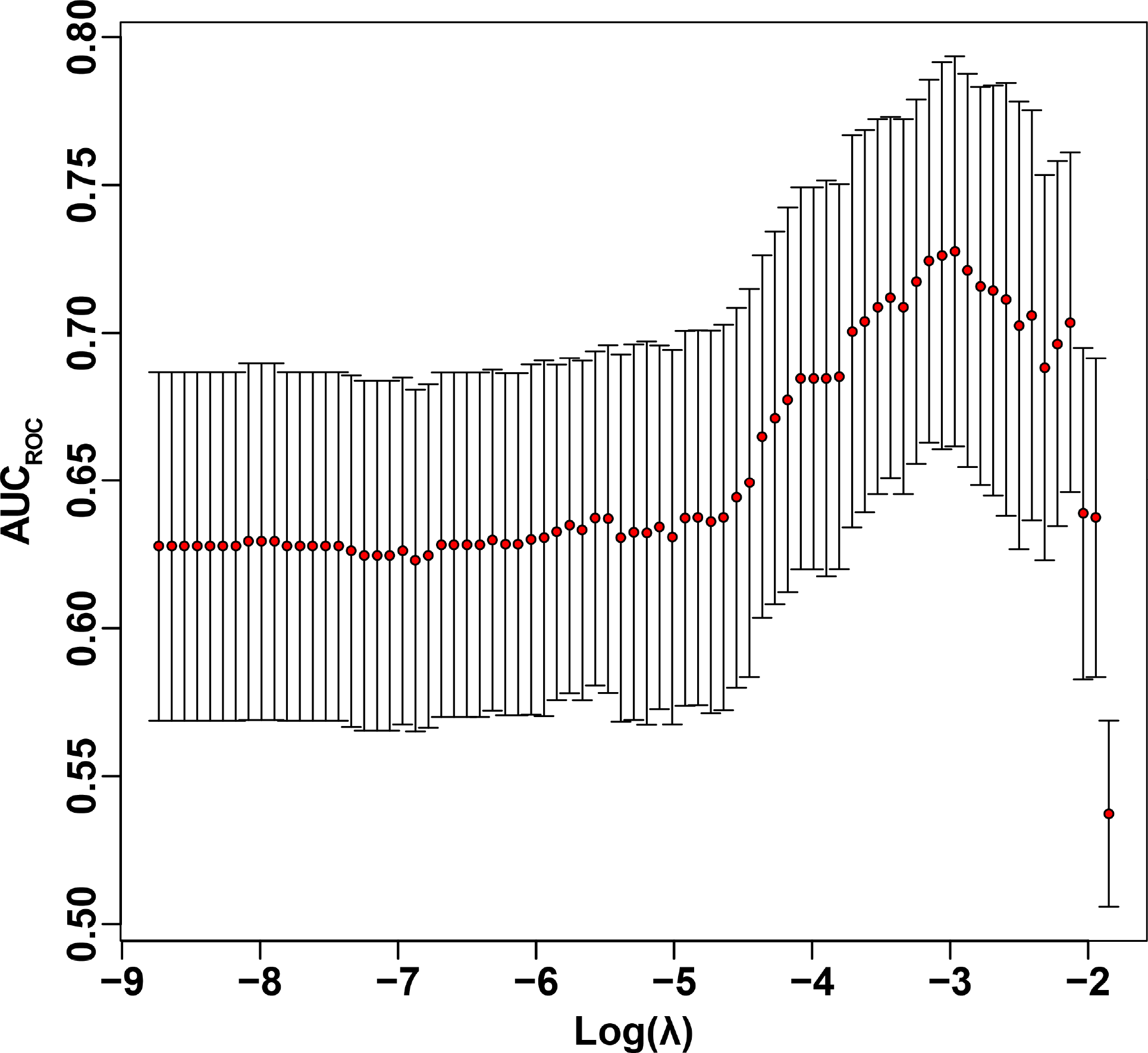

Supplement: S3 Fig — Tuning process maximized 5-fold cross-validated AUCROC as a function of log(λ). (TIF) [file pcbi.1009144.s003.tif]
