# Supplementary material for: PEDF, a pleiotropic WTC-LI biomarker: Machine learning biomarker identification and validation
Source: PLoS Comput Biol. 2021 Jul 21;17(7):e1009144. doi: 10.1371/journal.pcbi.1009144 (PMC8328304; doi:10.1371/journal.pcbi.1009144)
Supplement: S1 Table — (DOCX) [file pcbi.1009144.s004.docx]

| **S1 Table: Overview of Analytes and Representative References** | | |
| --- | --- | --- |
|  | **Variable** (n=111) | **Reference** |
| **Clinical** | Hear Rate, Systolic Blood Pressure | [1-4] |
|  | BMI at WTC-HP | [1-4] |
|  | Age on 9/11/2001 | [1-4] |
|  | Exposure | [2,4-15] |
|  | GM-CSF, MDC, IP-10, G-CSF, sCD40L, IL-(1b, 1a, 1ra, 2, 3, 4, 5, 6, 8, 9,10, 12p40, 12p70, 13, 15, 17) Eotaxin, EGF, Fit3-Ligand, Fractalkine, GRO, MCP-1, MCP-3, MIP-1α, MIP-1β, VEGF, TGF-α | [10] |
|  | CRP, MPO, sVCAM-1, MIP-4 | [7] |
| **MMP** | 1,2 3,7, 8, 9, 12 13 | [11,16] |
| **TIMP** | 1, 2, 3, 4 | [11,16] |
|  | Amylin, C-peptide, Ghrelin, GIP, GLP-1, Glucagon, Insulin, Leptin, PP, PYY | [2] |
| **Lipid/RAGE Axis** | Dyslipidemia | [1-3] |
|  | sRAGE | [12] |
|  | LPA | [5] |
| **TNF** | -α, -β | [2,10] |
| **IFN** | -α2, -γ | [10] |
| **Ig** | G _1,2,3 4,_ A, E | [6] |
|  | M |  |
| **Apo** | AI, AII, CII, CIII, E | [7] |
|  | B |  |
| **sIL** | -2Rα, 1RII, -6R, 1RI, -4R |  |
|  | PEDF, Osteopontin, sICAM-1, FGP2, Prolactin, Complement C4, sESelectin, sEGFR, tPAI-1, Adiponectin, sgp130, sCD30, α1-Antitrypsin |  |
| **sTNF** | RI, RII |  |
|  | SAA, SAP, CA-125, MIF |  |
| **sVEGF** | R1, R2, R3 |  |
| The 580 metabolites were previously available.[15]  **APO** Apolipoprotein; **sIL** soluble interleukin; **IL** Interleukin; **Ig** interferon gamma; **MIP** macrophage inhibitory protein; **TNF** tumor necrosis factor; **MMP** matrix metalloprotein; **TIMP** tissue inhibitors of metalloproteinases. | | |

**REFERENCES**

1. Kwon S, Crowley G, Mikhail M, Lam R, Clementi E, Zeig-Owens R, et al. Metabolic Syndrome Biomarkers of World Trade Center Airway Hyperreactivity: A 16-Year Prospective Cohort Study. Int J Environ Res Public Health. 2019 Apr 26;16(9).

2. Naveed B, Weiden MD, Kwon S, Gracely EJ, Comfort AL, Ferrier N, et al. Metabolic syndrome biomarkers predict lung function impairment: a nested case-control study. Am J Respir Crit Care Med. 2012 Feb 15;185(4):392-9.

3. Kwon S, Crowley G, Caraher EJ, Haider SH, Lam R, Veerappan A, et al. Validation of Predictive Metabolic Syndrome Biomarkers of World Trade Center Lung Injury: A 16-Year Longitudinal Study. Chest. 2019 Sep;156(3):486-96.

4. Schenck EJ, Echevarria GC, Girvin FG, Kwon S, Comfort AL, Rom WN, et al. Enlarged pulmonary artery is predicted by vascular injury biomarkers and is associated with WTC-Lung Injury in exposed fire fighters: a case-control study. BMJ Open. 2014 Sep 29;4(9):e005575.

5. Tsukiji J, Cho SJ, Echevarria GC, Kwon S, Joseph P, Schenck EJ, et al. Lysophosphatidic acid and apolipoprotein A1 predict increased risk of developing World Trade Center-lung injury: a nested case-control study. Biomarkers. 2014 Mar;19(2):159-65.

6. Cho SJ, Nolan A, Echevarria GC, Kwon S, Naveed B, Schenck E, et al. Chitotriosidase is a biomarker for the resistance to World Trade Center lung injury in New York City firefighters. J Clin Immunol. 2013 Aug;33(6):1134-42.

7. Weiden MD, Naveed B, Kwon S, Cho SJ, Comfort AL, Prezant DJ, et al. Cardiovascular biomarkers predict susceptibility to lung injury in World Trade Center dust-exposed firefighters. Eur Respir J. 2013 May;41(5):1023-30.

8. Weiden MD, Kwon S, Caraher E, Berger KI, Reibman J, Rom WN, et al. Biomarkers of World Trade Center Particulate Matter Exposure: Physiology of Distal Airway and Blood Biomarkers that Predict FEV(1) Decline. Semin Respir Crit Care Med. 2015 Jun;36(3):323-33.

9. Weiden MD, Ferrier N, Nolan A, Rom WN, Comfort A, Gustave J, et al. Obstructive airways disease with air trapping among firefighters exposed to World Trade Center dust. Chest. 2010 Mar;137(3):566-74.

10. Nolan A, Naveed B, Comfort AL, Ferrier N, Hall CB, Kwon S, et al. Inflammatory biomarkers predict airflow obstruction after exposure to World Trade Center dust. Chest. 2012 Aug;142(2):412-8.

11. Kwon S, Weiden MD, Echevarria GC, Comfort AL, Naveed B, Prezant DJ, et al. Early elevation of serum MMP-3 and MMP-12 predicts protection from World Trade Center-lung injury in New York City Firefighters: a nested case-control study. PLoS One. 2013;8(10):e76099.

12. Caraher EJ, Kwon S, Haider SH, Crowley G, Lee A, Ebrahim M, et al. Receptor for advanced glycation end-products and World Trade Center particulate induced lung function loss: A case-cohort study and murine model of acute particulate exposure. PLoS One. 2017 Sep 19;12(9):e0184331.

13. Prezant DJ, Weiden M, Banauch GI, McGuinness G, Rom WN, Aldrich TK, et al. Cough and bronchial responsiveness in firefighters at the World Trade Center site. N Engl J Med. 2002 Sep 12;347(11):806-15.

14. Rom WN, Reibman J, Rogers L, Weiden MD, Oppenheimer B, Berger K, et al. Emerging exposures and respiratory health: World Trade Center dust. Proc Am Thorac Soc. 2010 May;7(2):142-5.

15. Crowley G, Kwon S, Haider SH, Caraher EJ, Lam R, St-Jules DE, et al. Metabolomics of World Trade Center-Lung Injury: a machine learning approach. BMJ Open Respir Res. 2018 September 4th, 2018;5(1):e000274.

16. Nolan A, Kwon S, Cho SJ, Naveed B, Comfort AL, Prezant DJ, et al. MMP-2 and TIMP-1 predict healing of WTC-lung injury in New York City firefighters. Respir Res. 2014 Jan 21;15:5.
